# Modalities of Left Ventricle Decompression during VA-ECMO Therapy

**DOI:** 10.3390/membranes11030209

**Published:** 2021-03-16

**Authors:** Juan Pablo Ricarte Bratti, Yiorgos Alexandros Cavayas, Pierre Emmanuel Noly, Karim Serri, Yoan Lamarche

**Affiliations:** 1 Montreal Heart Institute, Université de Montréal, 5000 Belanger Street, Montreal, QC H1T 1C8, Canada; jpricartebratti@gmail.com (J.P.R.B.); yiorgos.alexandros.cavayas@umontreal.ca (Y.A.C.); noly.pierreemmanuel@gmail.com (P.E.N.); karimserri@hotmail.com (K.S.); 2 Hôpital Sacré-Coeur de Montréal, 5400. Gouin Blvd. West, Montreal, QC H4J 1C5, Canada

**Keywords:** VA-ECMO, left ventricular distention, left ventricular assist device, left ventricular venting

## Abstract

Veno-arterial extracorporeal membrane oxygenation (VA-ECMO) is used to sustain circulatory and respiratory support in patients with severe cardiogenic shock or refractory cardiac arrest. Although VA-ECMO allows adequate perfusion of end-organs, it may have detrimental effects on myocardial recovery. Hemodynamic consequences on the left ventricle, such as the increase of afterload, end-diastolic pressure and volume, can lead to left ventricular (LV) distention, increase of myocardial oxygen consumption and delayed LV function recovery. LV distention occurs in almost 50% of patients supported with VA-ECMO and is associated with an increase in morbidity and mortality. Thus, recognizing, preventing and treating LV distention is key in the management of these patients. In this review, we aim to discuss the pathophysiology of LV distention and to describe the strategies to unload the LV in patients supported with VA-ECMO.

## 1. Introduction

The prognosis of cardiogenic shock is poor, with a mortality rate of almost 50%, even in specialized centers [1]. Mechanical support systems have been developed to try to improve outcomes of patients with cardiogenic shock by (a) providing circulatory support by increasing blood flow and mean arterial pressure and (b) reducing LV wall stress, stroke work and myocardial oxygen consumption by reducing ventricular pressure and volume. Extracorporeal membrane oxygenation (ECMO) was introduced in 1977 to support patients with severe respiratory failure and/or cardiogenic shock [2]. The extracorporeal circulation can supply oxygenated blood to organs, thereby preserving their function. It also enhances coronary blood flow and reduces the time taken for the restoration of circulation in patients with cardiac arrest [3].

However, ECMO has device-related disadvantages such as hemolysis, coagulation disorders and limited device durability. Furthermore, the efficacy of ECMO has been questioned with respect to the mechano-energetic status of the heart [4,5]. Indeed, while peripheral veno-arterial (VA)-ECMO can effectively provide circulatory support, it does not completely unload the LV in patients with severe LV dysfunction [4]. This may result in LV distention in certain circumstances and may require adjuvant strategies for LV unloading. In this review, we aim to discuss the pathophysiology of LV distention and to describe the strategies to unload the LV in patients supported with VA-ECMO.

## 2. Mechanisms of LV Distention

In peripheral VA-ECMO, deoxygenated blood is drained from the right atrium (through inferior or superior vena cava) and oxygenated blood is reinjected through a femoral, axillary, aortic or carotid arterial cannula. Blood in the ECMO circuit thereby bypasses the right ventricle (RV), the pulmonary circulation, the left atrium (LA) and the LV.

This setting provides a non-physiological retrograde blood flow leading to significant hemodynamic changes. The main adverse effects of ECMO on a failing heart are increased LV wall stress caused by increased afterload and/or insufficient blood drainage [5]. The decrease in transpulmonary blood flow reduces LV preload, but the retrograde blood flow in the aorta also increases the mean arterial pressure and the LV afterload. This significantly increases myocardial oxygen consumption. In the presence of severe LV dysfunction, the left ventricle cannot sustain such an increase in afterload resulting in a dramatic fall in LV stroke volume. In patients with profound impairment of myocardial contractility, the left ventricle may even completely stop ejecting. If the venous return of the patient exceeds the extracorporeal blood flow, some venous blood enters the RV, which ejects it through the pulmonary circulation and contributes to LA and LV filling. Other sources of blood return in the LV are pulmonary arteriovenous shunts, aortic regurgitation, the bronchial circulation, and the Thebesian veins. Despite adequate venous drainage with VA-ECMO, there is usually some blood flow through the pulmonary vascular bed and some blood return in the LA. In this context, both LV end-diastolic volume and pressure may progressively increase if there is no ejection. The pulmonary capillary wedge pressure (PCWP) increases, leading to pulmonary congestion. Furthermore, the LV distention increases LV wall tension and may compromise subendocardial coronary perfusion, further impairing LV performance. Finally, the combination of poor LV contractility and reduced or absent aortic valve opening in systole, may cause severe blood stasis in the LV and promote thrombus formation (Table 1) [6,7,8]. It remains to be determined whether the adverse consequences of VA-ECMO are less pronounced with a central cannulation (right atrium-ascending aorta), because of a more physiological antegrade flow in the aorta. 

## 3. Monitoring and How to Recognize LV Distention and Pulmonary Congestion in Patients with VA-ECMO

Risk factors for LV distention may include the underlying etiology of cardiogenic shock (myocarditis, post-cardiotomy, ischemic), the degree of myocardial dysfunction (need for cardiopulmonary resuscitation), low arterial pulsatility, aortic regurgitation, high mean arterial pressure, poor venous drainage, or pulmonary edema at the time of ECMO initiation. The gold standard to diagnose LV distention is the direct measurement of end-diastolic LV pressure, but it is seldom available outside of the operating room or the catherization lab. A pigtail can be inserted in the LV through peripheral arterial access to directly measure the LV filling pressure. 

Because this is rarely performed, clinicians need to rely on indirect signs or surrogates of LV distention. Overt pulmonary edema is the end-stage of LV distention and is usually easy to diagnose. Clinicians taking care of ECMO patients should be aware of more subtle clinical, radiological and echocardiographic signs of early LV distention. Reduced systemic arterial pressure pulsatility, for instance, may reflect the onset of LV distention. A pulse pressure below 10 mmHg is usually considered worrisome. The use of pulmonary artery catheters in patients on VA-ECMO patients offers a convenient way to monitor this phenomenon. Indeed, the progressive rise in pulmonary artery diastolic and wedge pressure should lead the clinician to suspect progressive LV distention and prompt the timely performance of echocardiography to confirm LV distention.

Echocardiographic findings of LV distention include increased LV end-diastolic diameter, increased E/E’ ratio, spontaneous echocontrast (“smoke”) or thrombus in the LV, and intermittent or absent opening of the aortic valve. Other signs of LV distention include: (a) evidence of pulmonary edema; (b) elevated LV filling pressures; (c) refractory ventricular arrhythmias; and (d) stagnant contrast in the pulmonary arteries on computed tomography or conventional angiography [9]. Patients with florid pulmonary edema, ventricular arrhythmia or evidence of complete stagnation of blood in the LV have clinically significant LV distention. This generally prompts immediate consideration for mechanical interventions to decompress the LV. More subtle signs of pulmonary edema on chest X-ray in combination with evidence of increased LV filling pressures may be categorized as subclinical LV distention. Chest ultrasound is also a useful tool to assess interstitial edema, pulmonary consolidation, or pleural effusions.

## 4. Therapeutic Strategies for LV Distention

LV unloading strategies can be passive or active. Passive LV unloading strategies include the use of inotropes, intra-aortic balloon pump, and atrial septostomy to lower LV preload and/or afterload. Active LV unloading generally refers to the direct suction of blood from the left-sided cavities through surgically or percutaneously inserted cannulas. The choice of LV unloading strategy should be guided by individual patient characteristics, taking into account center and physician experience. Some interventions like inotropic support, fluid restriction, diuresis and ultrafiltration may be effective [10,11], but may not suffice in some patients where more invasive interventions may be needed. We will review the different methods for LV decompression (Table 2) and the evidence that supports their use (Table 3).

### 4.1. Inotropic Support and Volemic Status Optimization

These are simple first-tier strategies designed to avoid LV distention and to promote LV ejection. Generally speaking, ECMO pump should be set to target the minimal extracorporeal blood flow required to maintain adequate organ perfusion. Similarly, the minimal MAP allowing adequate organ perfusion should be targeted to minimize LV afterload (usually between 60 and 75 mmHg). Finally, one should aim to keep the patient at the minimal volemic status, allowing adequate ECMO flow with inlet pressures below −100 mmHg and without drainage line chatter. This is to reduce LV preload as much as possible. After the initial 24–48 h, fluid removal is usually necessary, with diuretics or ultrafiltration. Clinicians should be aware that improper drainage cannula position may also lead to unnecessary fluid administration. Repositioning the cannula in that context may help avoid an unnecessary early fluid creep. If LV distention occurs despite these initial measures, low dose inotropic therapy can be considered with the objective of restoring minimal contractility, aortic valve opening and LV ejection. This has to be balanced against the increased myocardial oxygen demand, the change in peripheral vascular tone and the arrhythmic risk of inotropic therapy.

### 4.2. Intra-Aortic Balloon Pump

The intra-aortic balloon pump (IABP) is used in ECMO patients to reduce afterload via a “Venturi” effect, to promote aortic valve opening and prevent LV thrombosis. The use of IABP reduces central venous pressure, PCWP and pulmonary edema on chest X-ray [12,13]. In patients without VA-ECMO, the IABP augments coronary, cerebral and visceral blood flow. On peripheral VA-ECMO, however, by interrupting retrograde flow in the aorta during diastole, the IABP could compromise cerebral and spinal cord perfusion [14,15,16,17]. The use of IABP with VA-ECMO has shown conflicting results, some studies reporting improved survival with the use of IABP in combination with ECMO [18,19,20], while others report no difference with or without IABP [21]. However, three recent independent meta-analyses, by Russo [22], Kowalewski [23], and Al-Fares [24], demonstrated that the use of IABP was associated with improved survival in patients supported with ECMO. It has been demonstrated to be useful in all VA-ECMO indications, including post-cardiotomy and refractory cardiac arrest [18,19], mainly by reducing pulmonary congestion and PCWP, and by increasing ECMO weaning [18,21]. The main advantages of the IABP are its low cost, the familiarity and simplicity of its use, the short term and ease of insertion at the bedside, and its low complication rate. Insertion of an IABP should be considered in patients with regular rhythm and cardiogenic shock due to myocardial infarction or post-cardiotomy shock after cardiac surgery due to its effects on coronary and graft blood flow [14]. The IABP is usually inserted through a femoral artery, the one contralateral to the femoral arterial return cannula in patients on a femoro-femoral configuration. The IABP is an effective and easy way to prevent LV distention and lung congestion if inserted before or at the same time as the ECMO. If LV distention is already installed and pulmonary edema is present, the IABP might be insufficient to reverse the process.

### 4.3. Balloon Atrial Septostomy 

Using a trans-septal puncture, an iatrogenic left-to-right shunt can be created, allowing blood aspiration by the venous cannula placed in RA and consequent LA decompression. Atrial septostomy has been shown to reduce inotropic support and facilitate weaning from ECMO. The complication rate of this technique is around 10%. The resulting LA pressure drop is 15 mmHg on average [25]. Alhussein et al. reported a reduction in the need for inotropic support, a 71% improvement in LV function and 70% of successful ECMO weaning in an adult population [26]. Similar results have been reported in children [27]. Several brief reports with excellent results have been published [28,29,30]. The size of the shunt should be discussed during the atrial septostomy, with progressive dilatation from 1 to 2 cm as a fully vented LV can lead to a cul-de-sac effect with potential non ejecting LV and apical thrombosis. A progressive dilatation, with transesophageal echocardiography monitoring of the trans-septal gradient leads to adequate unloading without complete shunting of the left atrial return to the right atrium. Definitive closure of the shunt is feasible after patient recovery either by surgical or percutaneous approach [31,32]. The atrial septum defect needs to be closed at the time of LVAD or total artificial heart implantation. 

### 4.4. Impella

Impella (AbioMed, Danvers, MA, USA) is a nonpulsatile axial flow pump that is placed through the aortic valve, pumping blood from the LV into the ascending aorta. Importantly, unlike IABP therapy, Impella does not require ECG or pressure triggering, facilitating stability even in the setting of arrhythmias or electromechanical dissociation. There are three versions available: LP 2.5 and 5.0 that can deliver 2.5 L/min and 5 L/min of cardiac output, respectively and CP that can deliver 3.5 L/min of cardiac output. Impella LP 5.0 needs to be inserted by surgical cut down (22 Fr sheath), whereas the other devices can be inserted percutaneously in a catheterization lab. Severe aortic valve calcification or insufficiency contra-indicate its use because of the risk of embolization in stenosis and the futile recirculation in the regurgitant valve. First successfully used in a patient with fulminant myocarditis [33], the use of the Impella device has since greatly increased. It may be used to treat LV distention because it decreases LV diastolic diameter and pressure, as well as the PCWP. It also increases global systemic blood flow and reduces LV stasis on echocardiography and improves the imbalance between O_2_ consumption and delivery [34,35]. It also has favourable effects on pulmonary congestion and right ventricular performance [34,36]. In a recent large retrospective study, the use of Impella in combination with ECMO in patients with refractory cardiogenic shock was associated with a decreased mortality as compared to ECMO alone (47% vs. 80%, respectively) [37]. This strategy allowed successful bridging to recovery or next therapy (68% vs. 28%, respectively). The bleeding rate was similar between groups, but increased hemolysis and increased duration of mechanical ventilation were noted in the Impella group, perhaps due to survival bias. A recent study where VA-ECMO was used with Impella demonstrated that mortality remains high but below risk score predictions [38]. More recently, Schrage et al. showed in a matched retrospective study of 510 patients that the use of Impella plus VA-ECMO vs. VA-ECMO alone decreased the probability of death, despite increasing some complications such as bleeding, hemolysis, ischemic complications and renal replacement therapy [39]. In this group of patients, those who benefited the most were those who had the Impella implanted early.

In patients with severe LV distention or pulmonary edema refractory to IABP, direct aspiration of blood from the LV with Impella can reduce LV distention, reduce O_2_ imbalance, improve right ventricular performance and reduce pulmonary pressures [34,35,36], leading to a higher rate of recovery, bridging and survival [37]. In addition, since patients on ECMO require very minimal drainage from their LV, the smaller percutaneously inserted versions of the Impella can be used. Finally, when VA-ECMO cannot be weaned off because of persistent left ventricular dysfunction, the Impella can be used to downgrade the mechanical circulatory support as an LVAD. Although experience with the device has increased, the availability and cost of the device limits its use in this context. This strategy also allows better assessment of the right ventricular function before considering durable LVAD implantation. 

Using an Impella device or other temporary LVADs alone can be done to provide both circulatory support and LV decompression without the need for ECMO. This, however, is not suitable for all patients as VA-ECMO is the only modality providing complete heart-lung support with high systemic flows, as well as blood oxygenation and decarboxylation. The optimal choice between these modalities in patients with isolated LV dysfunction is debated and should be tailored to individual patient needs while taking into account the context (urgent vs. semi-elective), center experience, and costs.

### 4.5. Surgical Decompression Cannula

Unloading the LV can be achieved by placing a drainage cannula in any part of the right to left circulation: the pulmonary artery, the LA or the LV itself. Various techniques have been described for LA or LV decompression, including percutaneous [40] and surgical ones. Surgical approaches carry a significant bleeding risk and are more often used when patients are centrally cannulated. Various surgical techniques have been described, including direct LA or pulmonary venous drainage (sternotomy or thoracotomy), left anterior mini-thoracotomy to cannulate the LA through the third or fourth intercostal space [41], or direct LV vent placement through left mini-thoracotomy with trans-apical off-pump insertion [42], a minimally invasive by sub-xiphoidal approach [43], axillary [44] or femoral approach [45]. Tepper and colleagues compared direct LV unloading with connection with the venous circuit of ECMO versus Impella (N = 45) and found similar mortality but a better reduction in pulmonary diastolic pressure in the direct LV vent group [46]. In general, the ideal option will depend on patients’ comorbidities, available vascular accesses and clinical status. When central ECMO is used for post-cardiotomy shock, surgical cannulation of the right superior pulmonary vein can be easily achieved to drain the LA or LV. The cannula is then connected to the venous drainage limb of the circuit. This is the easiest and most direct LV venting strategy in the post-cardiotomy setting. Its accessibility, as well as its routine use during cardiac surgical procedures, make it a very reliable first-line option. The use of this approach has also been reported in children and in non post-cardiotomy shock with good outcomes [47,48]. Direct drainage of the pulmonary artery can also be performed intraoperatively when technical considerations contraindicate direct drainage of pulmonary veins. A pulmonary artery cannula could eventually be used to transition towards an isolated RVAD in patients with residual RV failure, but this is outside the scope of this review.

### 4.6. Percutaneous Decompression Cannula

In general, in patients on peripheral ECMO, percutaneous venting, whether transeptal or transpulmonary, is simpler and more convenient than surgical approaches. Recently, Na et al. described LA drainage through central venous access and trans-septal puncture, aspirating the contents of the atrium and taking it to the ECMO circuit [49]. They also showed that prophylactic insertion of the cannula into position compared to placement as needed has mortality benefits, although the sample was small. Also, prophylactic cannulation had a higher rate of successful bridging to heart transplantation or LVAD and improved survival rate. On the other hand, the rate of successful weaning from ECMO and duration of ECMO support were similar in both groups. Major complications of the LA septostomy cannula technique include cardiac perforation during the insertion and subsequent cannula dislodgement. Percutaneous transpulmonary venting can also be achieved by the insertion of a 10 to 15 French catheter via the right internal jugular vein into the pulmonary artery [50,51].

## 5. Discussion

Refractory cardiogenic shock has led to the use of ventricular assistance. In the acute setting, VA-ECMO is increasingly being used [52], as it quickly restores end-organ perfusion and allows more time for a therapeutic decision: recovery, bridging or heart transplantation. The management of patients on VA-ECMO is challenging because a variety of complications may occur. One important problem is that VA-ECMO increases LV afterload in patients with often already impaired LV function, causing reduced forward flow with blood stasis, LV distention, increased filling pressures, and pulmonary edema. Incidences of LV overload up to 70% have been described in patients on ECMO with potential detrimental effects on morbidity and mortality [37,53]. 

**Figure 1:**
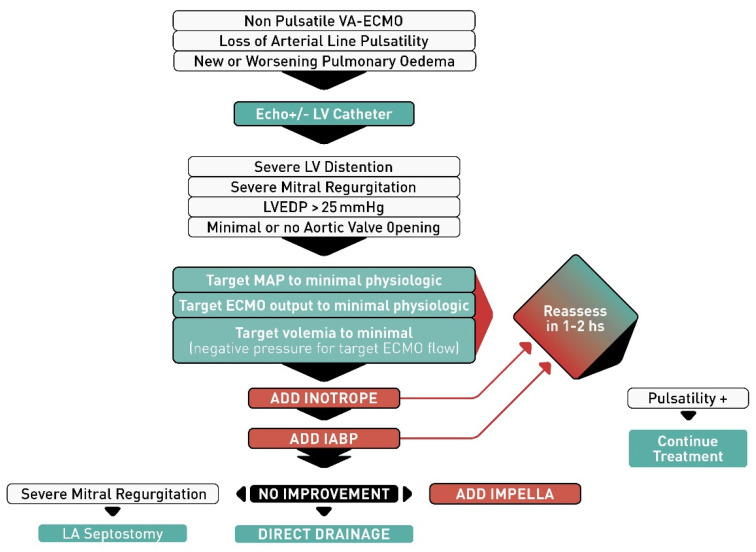
Algorithm proposed for left ventricular venting in patients with veno-arterial extracorporeal membrane oxygenation (VA-ECMO). Abbreviations: LV, left ventricular; LVEDP, left ventricular end-diastolic pressure; MAP, mean arterial pressure; IABP, intra-aortic balloon pump; LA, left atrial.

First-line treatments of LV distention include optimal management of the volume status and inotropes as an initial attempt to improve LV contraction and opening of the aortic valve. In general, a pulsatile arterial line tracing virtually rules out LV distention, because the heart has the strength to open the aortic valve and generate systolic and diastolic pressures, expelling some blood in each beat. Conversely, the loss of a pulsatile arterial line tracing or pulmonary edema should be viewed as an emergency and lead to a reassessment of the patient’s mechanical support configuration. This can be accomplished by echocardiography and/or LV catheterization, which may be performed at the bedside or in a cardiac catheterization lab with the insertion of a peripheral retrograde pigtail catheter into the LV cavity to document LV pressure. An elevated LV end-diastolic pressure is an indication to upgrade the venting strategy. We propose a decision algorithm for the management of this condition (Figure 1). We propose the use of inotropes and IABP as second line therapies if optimization of ECMO flows, vasoactive drugs and fluid therapies fail to improve the LV distention. If inotropes and IABP fail, more invasive active unloading therapies should be considered. In the presence of dynamic/functional MR secondary to LV distention, atrial septostomy or active left atrial drainage through a trans-septal cannula could be great choices as they can immediately decrease LV pressure both in systole and diastole. In the absence of mitral regurgitation, LA decompression can only decrease diastolic pressure, and only if the mitral valve opens in diastole. If LV diastolic pressure always exceeds LA pressure, the valve may not open and LA drainage may not be as efficient in decompressing the LV.

Intensivists, cardiologists and surgeons should collaborate to determine what type of physiology of extracorporeal circulation is present in every patient and use a stepwise approach to minimize complications and maximize benefit with this complex technology. A complete assessment of all parameters needs to be performed, including arterial pressure and its waveform, evidence of LV ejection, heart rate avoiding tachy or bradyarrhythmias, temperature, oxygen saturation and mechanical ventilation parameters according to the oxygen, acid-base analysis and volume and pressure status in the ventilator. In addition, daily echocardiograms for LV dimension measurements, assessment of aortic valve opening and the presence of thrombus are needed. Each case may need a different and individualized approach to LV decompression. 

In conclusion, left ventricular decompression is commonly needed in patients with VA-ECMO due to its effect on afterload. Close monitoring pulmonary artery and systemic pressures (mean and pulse), echocardiography, and X-ray may help identify early signs of LV distention and prompt quick optimization of medical management, to avoid severe pulmonary edema and the need for invasive decompression strategies. Several strategies are currently available to ECMO teams when faced with a patient with LV distention. Definitive trials confirming the superiority of one decompression technique over another are unavailable. A single decompression strategy is likely not optimal for any center as different clinical scenarios may benefit from different options. Also, more data regarding hemodynamic, physiology and physiopathology on LV decompression methods are needed.

## Figures and Tables

**Table 1:** Summary of hemodynamic effects of veno-arterial extracorporeal membrane oxygenation (VA-ECMO) leading to left ventricular (LV) distention.

Parameters	Consequences
LV Preload	↓
LV Afterload	↑
LV End-Diastolic Volume and Pressure	↑
Myocardial Oxygen Consumption	↑
LV Stroke Volume	↓

**Table 2:** Comparative effects of venting modalities.

	Modality	Advantages	Disadvantages
Passive LV Unloading	Inotropes	Simple, cheap, first gesture, no “instrumentation”	↑ Myocardial O_2_ consumption, ↑ risk or arrhythmias, discrete effect on venting
	IABP	Familiarity and simplicity of its use, ease and bedside insertion, low complication rate, enhances coronary circulation	Partial unload, limb ischemia, requires regular rhythm, contraindicated in aortic regurgitation and aneurysm
	Atrial Septostomy	Aspiration of blood through the RA-LA, if severe mitral regurgitation direct unloading of the LV	Risk of perforation/damage of neighbouring structures, stenting malposition, nephrotoxic contrast use, requires “resolution” after patient improvement
Active LV Unloading	Impella	Direct unload, trigger not required, further enhances systemic blood flow	↑ Risk of hemolysis, bleeding, and thrombosis, limb ischemia, contraindicated in aortic regurgitation and aneurysm, Impella 5.0 requires surgical insertion,
	Surgical decompression cannula	Direct unload	Insertion technique complications, if catheter placed in pulmonary artery lung ischemia may occur. Surgical approach requires sternotomy or mini-thoracotomy
	Percutaneous decompression cannula	Direct unload	Insertion technique complications

IABP, intra-aortic balloon pump; LV, left ventricular/left ventricle; RA-LA, right atrium-left atrium.

**Table 3:** Selected evidence for the use of LV unloading strategies.

Study	Year	Indications	No. Patients ECMO	No. Patients ECMO + Unload	Weaning ECMO (%)	Weaning ECMO + Unload (%)	Mortality ECMO (%)	Mortality ECMO + Unload (%)	Reference
IABP									
Aso et al.	2016	Any CS	1046	604	685 (65.5)	505 (83.6)	650 (62.1)	287 (47.5)	doi:10.1097/ccm.0000000000001828
Lin et al.	2016	Any CS	227	302	NR	NR	110 (48.5)	144 (47.7)	doi:10.1038/srep23838
Bréchot et al.	2018	Any CS	155	104	NR	NR	92 (59.4)	45 (43.3)	doi:10.1177/2048872617711169
Tepper et al.	2018	Any CS	30	30	16 (53)	20 (64)	22 (73)	15 (50)	doi:10.1097/MAT.0000000000000788
Doll et al.	2004	Post-cardiotomy shock	75	144	32 (42.6)	101 (70)	62 (82.6)	105 (72.9)	doi:10.1016/s0003-4975(03)01329-8
Wang et al.	2013	Post-cardiotomy shock	46	41	NR	NR	31 (67.4)	13 (31.7)	doi:10.1371/journal.pone.0063924
Impella									
Pappalardo et al.	2017	Any CS	42	21	16 (28)	10 (48)	21 (74)	10 (48)	doi:10.1002/ejhf.668
Patel et al.	2018	Any CS	36	30	16 (44)	21 (70)	28 (78)	17 (57)	doi:10.1097/mat.0000000000000767
Fiedler et al.	2018	Any CS	47	12	NR	NR	25 (53.2)	5 (41.6)	doi:10.1053/j.jvca.2018.05.019
LV Cannula									
Schmack et al.	2017	Any CS	28	20	NR	NR	21 (75)	9 (45)	doi:10.7717/peerj.3813
Comparison between Methods									
**Study**	**Year**	**Indications**	**Weaning Success ECMO + IABP (%)**	**Weaning Success ECMO + Septostomy (%)**	**Weaning Success ECMO + LV Cannula (%)**	**Mortality ECMO + IABP (%)**	**Mortality ECMO + Septostomy (%)**	**Mortality ECMO + LV Cannula (%)**	**Reference**
Hasde et al.	2020	Any CS	11 (55)	9 (52.9)	7 (43.7)	11 (55)	8 (47.1)	9 (56.3)	doi:10.1093/icvts/ivaa284

CS, cardiogenic shock; ECMO, extra-corporeal membrane oxygenation; IABP, intra-aortic balloon pump; LV, left ventricular; NR, not reported.

## Data Availability

Not applicable.
